# The Benefit of Anti-Inflammatory and Renal-Protective Dietary Ingredients on the Biological Processes of Aging in the Kidney

**DOI:** 10.3390/biology7040045

**Published:** 2018-09-29

**Authors:** Kiran S. Panickar, Dennis E. Jewell

**Affiliations:** Science & Technology Center, Hill’s Pet Nutrition, Inc., Topeka, KS 66617, USA; dennis_jewell@hillspet.com

**Keywords:** GFR, nephron, cytokines, SDMA, microbiome

## Abstract

One of the significant organ systems which decline in aging is the kidney. While the causes of age-associated decline in renal function are likely multifactorial, oxidative stress and inflammation are hypothesized to play important roles in the structural and functional changes of the kidney. During aging there is a general decline in the glomerular filtration rate (GFR), a primary measurement used to assess kidney function. Inflammation and oxidative stress have been hypothesized to have a significant detrimental effect on renal function in aging and this may be attenuated by renal protective dietary ingredients. These dietary ingredients may affect renal function directly or through a microbiome-mediated secondary product. Likewise, structural changes including renal tubular atrophy, interstitial fibrosis, and glomerulosclerosis have all been described in aging. Such detrimental changes may benefit from dietary ingredients that may delay or attenuate the occurrence of such changes. This review will describe the physiology and pathophysiology of aging in renal function with an emphasis on dogs and cats that develop a decline in kidney function naturally. In addition, the varying biomarkers of health and renal dysfunction will be discussed. Finally, we will evaluate the aid in the management of this normal decline through dietary intervention in animal models.

## 1. Introduction

Kidneys play an important role in the functioning of the body including filtering waste products and removal of toxins, the control of fluid osmolality, acid-base balance, controlling blood pressure, and even synthesizing erythropoietin to help stimulate production of erythrocytes in the bone marrow, when required. The functional unit of the kidney is a nephron which is composed of a renal corpuscle and a renal tubule. The renal corpuscle consists of capillaries called a glomerulus and Bowman’s capsule and the renal tubule extends from the capsule. Generally, a higher number of nephrons is associated with better renal function or functional capacity and a healthy adult kidney contains between 0.7 and 1.8 million functional nephrons in each kidney [1]. The process of urine formation begins when blood is filtered as it passes through the different layers in the kidney. Briefly, the stages of urine formation in the kidney involve filtration, reabsorption, and secretion. The glomerulus plays a key role in the initial filtration from where the filtrate passes through different segments of the nephron including the proximal convoluted tubule, loop of Henle, distal convoluted tubule, and the collecting duct.

Changes in renal structure (reduction in mass) and function (glomerular filtration rate (GFR)) accompany advancing age [1,2,3]. The number of tubular cells and glomerular cells decreases significantly during aging in humans and the number of glomerular tufts per unit area also decreased significantly with advancing age [4]. While several associated functional changes take place with aging, including a decline in renal blood flow, and sodium clearance, and the ability to maximally concentrate urine, this article will focus more on processes associated with the glomerular filtration rate, a generally accepted important assessment of kidney function. A decline in GFR has also been reported in cats and dogs with aging [5,6]. Such reduction in renal function with aging is likely multi-factorial [7] but an increase in inflammatory processes has been hypothesized to play an important role in the decline of renal function [8,9].

There is evidence to indicate that glomerulosclerosis, tubular atrophy, and interstitial fibrosis, all increase with aging, as assessed in autopsied tissues [10,11] and in tissues obtained from renal biopsies [12]. These pathophysiological changes can affect the whole kidney GFR [13]. GFR declines progressively with age by about 1 mL/min per year [14], although this can vary greatly depending on whether kidney aging progresses in the presence or absence of co-morbid health conditions. A reduced GFR with aging has been hypothesized and such reduction has been attributed to physiological aging in the absence of co-existing conditions such as kidney damage or other co-morbidities [13,15]. Renal inflammation is an important contributor to reduced GFR. Consistent with this, several studies have shown an association between higher pro-inflammatory markers and decreased GFR. However, the relation between GFR and inflammation is complex. Costello-White et al. [16] reported that in a study with 382 middle and older-aged adults in Japan and 1188 participants in the U.S. from the Midlife in the United States project (MIDUS), inflammatory markers including Interleukin-6 (IL-6), a cytokine, and C reactive protein (CRP), an acute-phase protein, were associated with GFR but in a biphasic manner depending upon the age of the participant. Low-grade inflammation tended to increase the GFR in adults below 49 years of age, but worsened the age-related decline in GFR in older participants over 65 years of age. In a study of 1590 middle-aged people from the general population without prevalent kidney disease, diabetes, or cardiovascular disease, after a median 5.9 years, 1296 persons were included in a renal follow-up study [17]. In this study higher baseline levels of high sensitivity C-reactive protein (hsCRP), but not tumor necrosis factor receptor 2 (TNFR2), a membrane receptor that also binds TNF-α, were associated with an accelerated age-related decline in measured GFR in the general population. Interestingly, Nerpin et al. [18] reported that cytokine-mediated inflammation was involved in the early stages of impaired kidney function in the elderly, but that cyclooxygenase-mediated inflammation did not appear to play a role at this stage in 647 men with a mean age of 77 years. Whether specific inflammatory proteins and pathways are involved in certain stages of renal dysfunction are not clear. However, it is a possibility. Nevertheless, these studies indicate that aging is associated with a decline in GFR and inflammation is one important factor that contributes to decreased renal function. 

Aging is associated with a decline in renal function in companion animals as well including dogs and cats. In addition, the renal morphology of dogs is very similar to human kidney [19]. One advantage of studying companion animals is that they may represent a model of naturally occurring human disease [20]. It has been long observed that there is an increased incidence of renal disease as pets age. Symmetric dimethylarginine (SDMA), an isomer of asymmetric dimethylarginine (ADMA), is a biomarker of a decline in renal function. SDMA is similar to Cystatin C, a polypeptide that is also useful as a marker of renal function in cats [21], but SDMA has the advantage that it is not influenced by thyroid status [22] which has been shown to influence Cystatin C [23]. In humans, higher body mass index (BMI) has been shown to be independently associated with high Cystatin C levels in the absence of any clinically recognized chronic kidney disease (CKD) [24] and high Cystatin C levels are also correlated with accumulation of epicardial adipose tissue, a cardiovascular risk factor independent of renal function decline [25]. Circulating levels of SDMA increase in dogs [26] and cats [27] with declining renal function resulting in SDMA elevation even earlier than creatinine. Also, it has the advantage over creatinine that the lean body mass decline, often observed with aging, does not influence the concentration of SDMA as it does with creatinine [27]. Using creatinine and SDMA as markers of renal decline, approximately 45% of geriatric dogs have values which have increased to a point of concern [28] while cats have more renal function decline with age than do dogs. Whether an elevation in serum SDMA is a reliable marker for early kidney dysfunction in humans is not known, but an area of research that warrants investigation in human studies. While the National Kidney Foundation’s Kidney Disease Outcomes Quality Initiative (KDOQI) recommends estimating GFR and screening for albuminuria in patients with risk factors for chronic kidney disease, including diabetes, hypertension and aging (>60 years), the results from animal studies offer important alternatives to explore to early diagnose CKD. 

In humans, there also is a decrease in renal function associated with aging [29]. In unsupplemented humans with chronic kidney disease (CKD) renal decline was associated with both a decrease in circulating vitamin C [30] and vitamin E [31]. In dogs, supplementation of n-3 long chain polyunsaturated fatty acid (n-3 LCPUFA) and antioxidants was associated with attenuation in the decline of renal function [32]. Therefore, an increased supplementation of antioxidant ingredients and with n-3 LCPUFA are reasonable dietary interventions in foods designed for aging pets. Regarding renal function and aging the control of macronutrients, specifically protein and phosphorus are worthy of discussion. Because the kidneys are the main route of phosphorus excretion, declining function may result in phosphorus retention and significant health consequences. It is well established that the control of dietary phosphorus is helpful as an aid in the management of declining renal function [33,34]. Dietary phosphorous restriction has been recommended to support renal function during aging and kidney disease in pets as well as in humans with early stage kidney disease [35]. Maintaining phosphorus intake in excess of the minimum optimal level while avoiding excess is a reasonable dietary goal for aging pets. Dietary protein concentration for aging is more controversial. First there is the need for protein concentration to be above that which is optimal for all body function which must be met for optimal health. Optimal protein intake allows for maintenance of lean body mass, and a multitude of protein requiring functions (e.g., circulating protein, immune response, optimal skin and coat quality). On the other hand protein intake above that for optimal health is of no value and excess protein must be excreted either through breakdown and nitrogenous waste filtered through the kidney, or it is not absorbed and available to the microbes through proteolytic catabolism and fecal excretion. Both of these may have negative effects on renal health in aging—if absorbed especially in the presence of reduced renal function it is likely to promote the progression of reduced renal function [36] or may be a component of some of the negative metabolic consequences of aging [37]. High protein intake was associated with increased interstitial inflammation in rats with surgically induced chronic renal failure [38]. Also, dietary protein was associated with immune-mediated renal damage in models of renal disease [39,40]. Dogs and cats tend to have reduced renal function with aging which when monitored by creatinine concentration is unfortunately masked by changes in lean body mass for the dog [41] and cat [27]. In humans, based on the CKD stage, if significant protein is lost during dialysis then the intake of proteins to replace the lost protein is recommended depending on other health conditions. One important contributor to a decline in kidney function in aging humans is obesity. A study of 2489 individuals with a mean age of 74 ± 3 years showed a significant interaction between visceral abdominal fat and CKD with regard to a decline in kidney function [42]. Importantly, several co-morbidities in the aging population make the elderly more susceptible to kidney dysfunction including hypertension and diabetes (for review see [43]). There is also evidence to indicate that aging greatly increases the risk of the progression of acute kidney injury to CKD and chronic inflammation is hypothesized to be as an important contributor for such progression [44]. 

## 2. Immune System

Immunosenescence is a contributor to the observed reduction in health during aging. Due to cellular changes that occur in the aging kidney many functional changes can occur, including an increased tendency for apoptosis, reduction in epithelial cell proliferation, some changes in expression of growth factors (decrease in epidermal growth factor (EGF), insulin-like growth factor (IGF), and vascular endothelial growth factor (VEGF) but an increase in transforming growth factor-β (TGF-β)), and also changes in stem and progenitor cell functions [45]. In general, it appears that aging does not reduce the total number of neutrophils but does result in a reduction of function such as chemotaxis and phagocytosis [46]. It is possible that membrane fluidity changes mediate many of these dysfunctional signaling pathways that are found in aging neutrophils [47]. These pathways are naturally influenced by the abundance of mRNAs specific for neutrophil activity. For example, most mammalian neutrophils kill bacteria that are phagocytized through oxygen dependent mechanisms that rely on myeloperoxidase (MPO), an enzyme predominantly expressed in neutrophils. Also, neutrophil migration must be initiated and coordinated which is influenced by the formation and breakage of carbohydrate-selectin bonds. A leukocyte glycol-protein (L-selectin) is involved with the binding and rolling of leukocytes into sites of inflammation [48]. The chemokines such as IL-8 and their receptors such as IL-8R are involved with the interactions of neutrophils and the induction of migration as well as also enhancing phagocytosis and reactive oxygen species generation [49,50,51]. Hall et al. [52] reported a loss of neutrophil function and the simultaneous decrease in L-selectin and IL-8R mRNA in older dogs when compared to young, indicating that older dogs have reduced innate immune response. Also, healthy aging adults have higher circulating levels of cytokines including IL-6, IL-8, IL-10, and TNF-α [53]. Several of these circulating cytokines including TNF-α and IL-6 are higher in patients with chronic renal failure [54,55] and this can occur even in the absence of aging. It is conceivable that indicating higher levels of circulating cytokines in aging can affect kidney function. Measurement of urinary cytokines may be another indicator of assessing renal health. In humans, urinary concentrations of IL-6, IL-8, monocyte chemoattractant protein-1, interferon-gamma-inducible protein (IP-10), and macrophage inflammatory protein-1δ) were higher in patients with microabuminuria and a decline in renal function when compared with patients with normoalbuminuria and stable renal function [56]. In cats with CKD, levels of cytokines including IL-8 and transforming growth factor-β1 (TGF-β1) were significantly higher in the urine of CKD cats when compared to controls but significantly lower levels of vascular endothelial growth factor (VEGF) were reported [57]. While more work is needed to determine the value of assessing cytokines in urine as well as determining if any particular urinary cytokine is more indicative than others of CKD, the value of such noninvasive biomarkers of CKD is undeniable.

A significant effector of aging in the immune system, as discussed above, is apoptosis which is achieved through a cascade of intracellular proteases—the caspases [58,59]. The first such protein to be identified was caspase-1 also known as the interleukin-1-converting enzyme (ICE). This protein processes pro-IL-1β to yield active interleukin-1β [60], a cytokine which inhibits the expression of FAS- mediated apoptosis [61] and plays an essential role in inflammatory cell activation [62]. It is clear that the aging process is strongly influenced by IL-1β and makes it a target for nutritional intervention. The decrease of interleukin converting enzyme (ICE) mRNA as dogs aged was shown by Hall et al. [52]. Interestingly, in humans undergoing dialysis, Th-1 lymphocytes have a decreased expression of the anti-apoptotic molecule Bcl-2, which makes the Th-1 cells more susceptible to apoptosis [63]. In patients with uremia, in advanced CKD, there is a reduction in the total number of T-lymphocytes and an imbalance in the ratio of Th-1/Th-2 cells due to a higher rate of apoptosis in Th-1 cells is hypothesized to contribute to altered immunity in CKD patients [64]. Nutrition can aid in aging through changing the mRNA and immune system functions and may have a direct effect on influencing kidney health. It has been shown that dietary antioxidants, coupled with behavioral enrichment, enhance immune function as shown by neutrophil phagocytosis in older beagles [65]. In that intervention the dietary ingredients included were a milieu of antioxidants (Vitamins E, C, and alpha lipoic acid), combined with flavonoids and carotenoids from vegetables, as well as the mitochondrial cofactor L-carnitine. When this enhanced nutrition was combined with behavioral enrichment there was an enhanced phagocytosis in elderly Beagles [65]. Subsequently, it was shown that foods rich in Vitamins E and C with or without the addition of fish oil were effective in increasing neutrophil mediated cell killing and increased mRNA for IL-8R and L-selectin. This was coupled with a decrease in the pro-inflammatory TNF-α mRNA [66].

Ingredients including bioactive extracts from herbs and plants have been shown to have renal-protective effects by decreasing the pro-inflammatory cytokines in animal models of renal dysfunction or in cell culture studies. These studies are important because diets or nutrition supplemented with such extracts may provide beneficial effects in reducing renal dysfunction. Extracts of *Hydrangea paniculata* (HP), a traditional Chinese medicinal plant, containing the bioactive coumarin, exerted renoprotective effects in a lipopolysaccharide (LPS)-induced mouse model of septic acute kidney injury [67]. The anti-inflammatory effects of HP were through inhibiting the infiltration of neutrophils and macrophages into kidney tissues, as well as through reducing the production of cytokines and chemokines. Grape seed procyanidin extract (GSPE) attenuates arsenic trioxide (As_2_O_3_)-induced renal inflammation in mice by reducing cytokine production [68]. In rats, the aqueous leaf extract of *Madhuca longifolia*, a deciduous tree, showed a significant protective effect in renal toxicity induced by diclofenac, with a concomitant reduction in serum cytokines including IL-6, IL-1β and TNF-α [69]. Fruit extract of *Withania coagulans* (Solanaceae), with the bioactive withaferin A, significantly protected rat kidneys from oxidative stress and free radical-induced DNA damage in cisplatin-induced nephrotoxicity in rats [70]. In addition, there was also a decrease in IL-1β, IL-6, and TNF-α in the kidney tissues of cisplatin-treated rats when compared to controls. These studies indicate that ingredients with anti-inflammatory properties may be beneficial.

## 3. Inflammation

Inflammation is a well-known and well-studied component of many of the natural aging consequences and the renal decline associated with aging. The inflammatory state in aging is hypothesized to be a risk factor for accelerated decline in renal function even in the absence of co-morbid conditions like diabetes and blood pressure in humans [71]. One of the new techniques of evaluating the effects of dietary changes on these processes is that of metabolomic profile analysis. Metabolomics allows the study of individual metabolites in a cell, tissue, or blood which are the end-products of various cellular processes. This analysis allows measurement in changes in low molecular weight metabolite populations and how this balance may be changed by dietary intervention [72]. These small molecules may themselves be active components in the processes or may be surrogate biomarkers for shifts in metabolism associated with the dietary intervention [73]. The aging effect on circulating lipids and the intervention through dietary manipulations is an ongoing and fruitful area of research. For example, we have shown that there are specific age associated changes LCPUFA acids in dogs [74]. This is similar to the declining n-3 and n-6 LCPUFA reported in humans where these changes are associated with changes in circulatory inflammatory markers [75]. In general the n-3 LCPUFA are anti-inflammatory as well as immunodulating [76]. At a dietary concentration sufficient to allow changes in membrane lipid incorporation eicosapentaenoic acid [EPA, 20:5 (n-3)] and docosahexaenoic acid [DHA, 22:6 (n-3)] influence the fluidity and physical nature of cell membranes as well as membrane protein-mediated responses [77]. In general the LCPUFA EPA may protect against excessive inflammatory reactions and is potentially in balance with the eicosanoids derived from the n-6 LCPUFA arachidonic acid [AA, 20:4 (n-6)]. There is strong evidence that n-3 LCPUFA have a beneficial effect on clinical symptoms associated with excessive inflammation and renal function [78,79]. In dogs, we have shown that there is a beneficial effect to dietary LCPUFA in the nutritional aid in management of inflammation allowing, reduced pain and increased movement [80,81,82] as well as enhanced renal function [27]. There is an overall reduction of biomarkers of inflammation in dogs when dietary n-3 LCPUFA are increased, suggesting that these nutrients would benefit any time there is chronic inflammation associated with aging [83]. Dietary intake of PUFA in humans is hypothesized to delay the progression of CKD.

These changes associated with the LCPUFA may be accentuated by the declining concentration of carnitine associated with age. Carnitine performs a required role in mitochondrial oxidation of long chain fatty acids as a cofactor in specialized acyltransferases [84]. To summarize L-carnitine is required in the transportation of long chain fatty acids from the cytosol to the mitochondria for beta-oxidation. The by-product of beta oxidation, acetate then provides energy through the normal metabolism of the citric acid cycle. Under normal conditions L-carnitine is not considered to be a limiting nutrient. However, the addition of dietary L-carnitine can offset the normal decline of circulating carnitine associated with aging [74] and increase lean body mass in dogs [85] and cats [86]. The supplementation of L-carnitine in humans has been shown to reduce C-reactive protein a recognized biomarker for inflammation [87] as well as aiding in the management of renal failure [88]. It has been suggested that L-carnitine is a precursor of Trimethylamine N-oxide (TMAO) which is a toxic compound that accelerated atherosclerosis in mice [89]. In the same study, plasma L-carnitine levels, in human subjects undergoing cardiac evaluation, predicted increased risks for both prevalent cardiovascular disease (CVD) and incident major adverse cardiac events (e.g., myocardial infarction) in subjects with high TMAO levels. However, the relationship between TMAO levels and early atherosclerosis in humans is not clear. For instance, Randrianarisoa et al. [90] reported that in the subjects who participated in the Tubingen Lifestyle Intervention program, during the lifestyle intervention most cardiovascular risk parameters improved but no change in mean levels of TMAO. In the context of kidney function, it has been suggested that TMAO may not be anything more than a bystander in the development of kidney disease [91].

A decrease in the levels of Vitamin D3 (Cholecalciferol) in the form of 25-hydroxyvitamin D (calcidiol) or 1,25-dihydroxyvitamin D (Calcitrol) has been reported with aging although some studies have reported no change or even an increase (see [92] for review). Activated form of Vitamin D is formed through two hydroxylation processes. Initially Vitamin D is hydroxylated in the liver to produce 25-hydroxyvitamin D and subsequently hydroxylation of 25-hydroxyvitamin D occurs, predominantly in the proximal tubule of the nephron in the kidney, to form 1,25-dihydroxyvitamin D. Given that there is a decline in renal function in aging, it would be expected that supplementation of vitamin D would be beneficial. In support of this, subjects with severe renal dysfunction or early-to-late stages of CKD showed benefit when supplemented with cholecalciferol as assessed by an improvement in their vitamin D status [93,94]. However, there does not appear to be a consensus in the benefit of vitamin D supplementation in patients with CKD [95,96].

There is evidence, from rodent studies, that herbal extracts or bioactives from medicinal plants with anti-inflammatory effects are beneficial in reducing renal dysfunction. In a rat model of calcium oxalate urolithiasis, there was a decrease in monocyte chemoattractant protein-1 (MCP-1) in the renal homogenate of rats fed total flavonoids of *Desmosium styracifolium* (TFDS) when compared to un-treated rats [97]. In addition, TFDS also significantly reduced crystalluria and calcium oxalate crystal deposits in the kidney sections as compared to the untreated group. Terpen glycosides from Cortex Moutan, the root bark of *Paeonia suffruticosa*, reduced inflammatory cytokines including IL-6, MCP-1, and intercellular adhesion molecule 1 (ICAM-1), in a rat model of diabetic nephropathy [98]. In a folic acid-induced kidney injury model in mice, systemic administration of Tanshinone IIA, a diterpene and one of the abundant constituents of *Salvia Miltiorrhiza*, resulted in improved kidney function with a concomitant reduction in chemokine expression [99]. Tanshinone IIA also suppressed renal fibrosis and inflammation as assessed by a decrease in cytokines TNF-α, MCP-1, and the chemokine CXCL-1, in a rat model of CKD induced by nephrectomy [100]. These studies were conducted in animal models of renal injury in which inflammation was a major component. Nevertheless, the results from such studies indicate that herbal extracts or bioactives from such extracts may be useful in exerting an anti-inflammatory effect. Studies to assess the anti-inflammatory effects of herbal extracts on renal function in aging are warranted. 

## 4. Body Composition

Sarcopenia is a key feature associated with aging and is especially critical in the presence of CKD. It is characterized by myopenia (reduction in muscle mass), dynapenia (decreased muscle strength), and a resultant decline in muscle function. However, what is defined as muscle weakness may vary when age and gender-matched data are used for comparison. Sarcopenia is prevalent in patients with chronic kidney disease [101]. In a study of 148 adult patients with an estimated GFR <30 mL/min/1.72 m^2^, and CKD stage 3–5, there was a loss of lean body mass, especially appendicular skeletal muscle and this was significantly related to GFR decline [102]. It is an unfortunately common mammalian experience in aging to have both a loss of lean body mass and an increase in adiposity. This seems at least correlated with and may be causally connected with loss in renal function [5,103]. For instance, in humans a 1% loss of leg lean muscle per year was reported and in addition muscle strength loss was approximately three times that of muscle protein loss which was not restored by gain in muscle mass [104]. In another reported study where data from 10,734 adults with normal BMI from the Korean National Health and Nutrition Examination Survey (KNHANES) were analyzed, there was decreased lean mass concomitant with excess adiposity which was associated with CKD [105]. These studies indicate that sarcopenia and sarcopenic obesity may be common in later stages of CKD in humans. Pathogenic mechanisms underlying uremic sarcopenia are not clear but indoxyl sulfate, a uremic toxin, has been implicated as one pathogenic factor in CKD. Sato et al. [106] reported skeletal muscle atrophy in a mouse model of adenine-induced CKD associated with increasing levels of circulating indoxyl sulfate which also accumulated in the muscle cells of CKD mice. In the same study, using cell cultures, they also showed mitochondrial dysfunction in muscle cells with indoxyl sulfate. Subsequently Enoki et al. [107] also reported mitochondrial dysfunction in cell cultures with indoxyl sulfate. These studies indicate an important role of indoxyl sulfate in mitochondrial dysfunction and its potential role in sarcopenia. Another molecular mechanism by which indoxyl sulfate contributes to the development of sarcopenia is by inducing endoplasmic reticulum (ER) stress and the unfolded protein response (UPR) [108]. Zheng et al. [109] showed that in C2C12 mouse myoblast cells, indoxyl sulfate inhibited myoblast differentiation, induced the myotubular atrophy marker atrogin-1 protein expression, and may alter levels of XBP-1, a transcription factor and a key component of the ER stress response. Studies in companion animals also indicate that when cats [5] and dogs [6] are fed to maintain body weight, aging is associated with this change in body composition and this co-exists with a decline in renal function decline. This is in the presence of other classic markers of protein nutrition showing protein adequacy. Optimal dietary protein concentration will then be at a concentration during aging which maximizes body lean while controlling excess in a way that does not provide unwanted substrate for proteolytic microbiota metabolism or excessive nitrogenous waste for those pets with declining renal function. In order to provide optimum protein nutrition and avoid unwanted excess, protein quality is to be considered as well as protein quantity. Because dogs and cats do not have a true requirement for protein but rather for amino acids, protein quality is defined by the amino acid profile and digestibility.

Use of herbal extracts to alleviate some of the characteristics of sarcopenia has been investigated. Go-sha-jinki-Gan, a traditional Japanese herbal medicine, reduced the loss of skeletal muscle mass and ameliorated the increase in slow skeletal muscle fibers in senescence-accelerated mice (SAMP8) [110]. In an in vitro model of muscle cell proliferation using murine skeletal muscle myoblast cell line C2C12, hachimijiogan (HJG), a Japanese herbal medicine, significantly increased C2C12 cell number when compared to control [111]. Treatment of old mice with Jaeumganghwa-Tang, a traditional herbal formula composed of 12 medicinal herbs, improved muscle strength, increased skeletal muscle mass, and alleviated muscle damage [112]. While these studies were not conducted in humans or animals with renal dysfunction, it is conceivable that such herbal extracts or bioactives from such extracts may be useful in reducing effects of sarcopenia.

## 5. Microbiota

Characterization of the human bacterial population (microbiota) and its corresponding genome (microbiome) in aging reveals that shifts in the composition of the intestinal microbiota may lead to detrimental effects for the elderly host [113]. There is frequently a reduction of the proportion of the beneficial bacteria, an increase of non-beneficial bacteria which when combined are significant components of various negative changes with aging including a decline in renal function [114]. For instance, there is a decline in the beneficial bacteria such as bifidobacteria and an increase the number of facultative anaerobes [113,115]. Vaziri et al. [116] reported that patients with end-stage renal disease (ESRD) had higher gut bacterial diversity in some phyla than the control group and reported an increase in operational taxonomic units (OTUs), a method for grouping based on DNA sequences, from Brachybacterium, Catenibacterium, Enterobacteriaceae, Halomonadaceae, Moraxellaceae, Nesterenkonia, Polyangiaceae, Pseudomonadaceae, and Thiothrix families in ESRD patients and a decrease in Bifidobacteria and Lactobacilli. 

The postbiotics, metabolic products that result from microbial activity, associated with these changes may result in toxic microbial metabolites causing inflammation and oxidative stress which are often associated with disease progression and renal function decline. These toxic byproducts of fermentation have been shown to be related to constipation, mal-absorption and deterioration of the intestinal barrier integrity [117,118]. Several circulating metabolites of bacterial fermentation including trimethylamine N-Oxide, indoxyl-sulfate, p-cresyl-sulfate, and phenylacetylglutamine can all affect renal function adversely [119,120,121] and have been reported to be inversely associated with estimated GFR (eGFR) in subjects with minimal renal function decline [122] indicating that such metabolites may contribute to an eventual decline in renal function. 

A low-protein diet, if only to reduce the levels of uremic toxin, would appear to be beneficial in subjects with early stages of CKD. In support of this Black et al. [123] reported that in a longitudinal study with 30 non-dialysis CKD patients (stage 3–4) subjects on a low-protein diet for 6 months showed reduced levels of serum p-cresyl sulfate but such levels were increased in patients who did not adhere to the low-protein diet. An in vitro study has shown that canine fecal microflora are able to produce the beneficial post-biotics short chain fatty acids from fruits and vegetables [124]. This has been also shown in vivo in that foods containing fermentable fibers in dogs are beneficial in reducing enteric infection and increasing nutrient absorption [125]. Foods containing fruit and vegetable fiber sources have been shown to enhance renal function in aging pets while producing changes in the microbiota and post-biotics consistent with healthy aging and improved renal function [126]. When foods designed using this innovation in understanding of ingredients and aging were tested against controls, there was a significant benefit on renal function. There were significant mRNA decreases in interleukin-8receptor (IL-8R), interleukin converting enzyme (ICE), and myeloperoxidase (MPO) associated with these foods along with improvement in age related inflammation and skin and coat quality [6,127]. A significant practical benefit was shown in that there was an improvement in lean body mass in cats as compared to control foods with an increase in lean body mass while eating the test foods [128]. A benefit on renal function was shown in that it was enhanced in both dogs [126] and in cats [129]. There was also a significant enhancement in “renal positive” microbiota in that the pets fed the enhanced food increased healthy bacteria in the dog with an increase in the proportions of the genera adlercreutzia, oscillospira, phascolarcobacteria, faecalibacterium, and ruminococcus. This was accompanied by a reduction in the genera megamonas, salmonella, and peptostreptococcus, while in the cat feeding the feline enhanced food resulted in a significant increase in the genus bifidobacterium, which was accompanied by a decline in the proportions of the genera clostridium and eubacterium [130,131]. These microbiota changes were present in the pets with enhanced renal function as shown by a decline in circulating levels of SDMA, a marker of renal function decline. In summary, in order to provide the optimum balance and control proteolytic bacteria food should optimize the quotient of protein to carbohydrate and optimize the environment for renal protective microbiota.

## 6. Conclusions

**Figure 1:**
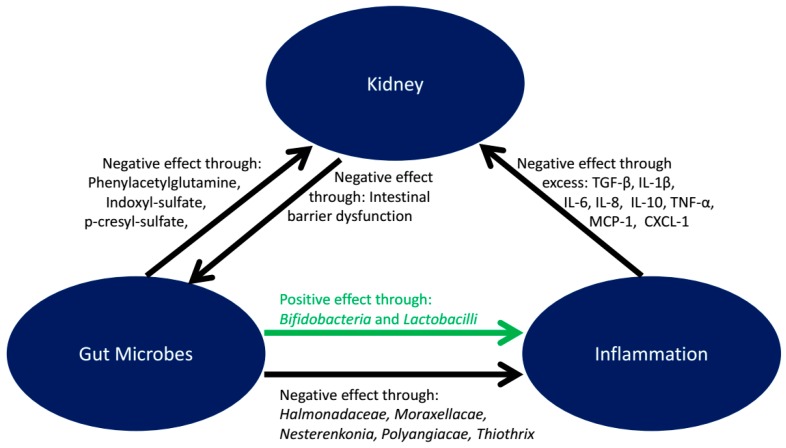
The relationship between gut microbes, inflammation, and kidney function. A schematic representation of the role of inflammation and gut microbes on kidney function. Inflammation in aging is mediated by increased levels of members from the cytokine and chemokine families. The negative effects of the gut microbial products including p-cresyl sulfate and indoxyl sulfate may induce kidney dysfunction directly or through these uremic toxin-mediated inflammation. The beneficial effects of the gut bacteria especially *Bifidobacteria*, on kidney function, may be through its effects by increasing the levels of anti-inflammatory cytokine IL-10. Dietary interventions to reduce such uremic toxins by modulating the gut microbiome may be an important area of research to attenuate the deleterious effects of aging. (Abbreviations: Transforming grow factor-β (TGF-β); IL-1-β (Interleukin-1β); IL-6 (Interleukin-6); IL-8 (Interleukin-8); IL-10 (Interleukin-10); TNF-α (Tumor necrosis factor-α); MCP-1 (Monocyte chemoattractant protein-1); CXCL-1 (Chemokine (C-X-C-motif) ligand 1)).

Inflammation is a major component of renal dysfunction in aging and may even exacerbate the decline in renal function. Despite inflammation contributing to renal dysfunction in aging, studies evaluating the benefits of anti-inflammatory ingredients are lacking. Although currently there is a significant use of small mammal models (rat and mouse), spontaneous development of renal insufficiency and disease in dogs and cats offers significant benefit over testing in experimentally induced disease. The applications and learnings from studies in healthy aging model populations can contribute to our understanding of beneficial best practices for therapeutic interventions in human populations. One example, as discussed above, is the use of SDMA as a marker of renal decline in cats that appears to have an advantage over using GFR. Whether elevation in serum SDMA is a reliable marker for early kidney dysfunction in humans is not known, but that is one area of research that warrants investigation in human studies. These animal model studies show that supplementation of a mildly protein-restricted food with fish oil, antioxidants, and fruits and vegetables to provide bioactive botanicals aids in the management of renal function decline and CKD. Using the innovations available to nutritional science today optimal foods can be produced that allow healthy aging through support of the foundational physiological underpinnings of renal function. There is a specific group of dietary interventions that build an optimum response to the challenges of aging on renal function including that of the immune system, inflammation, lean loss, and microbiota (see Figure 1). Reducing inflammation to improve renal function in aging should be an area to explore further and strategies to attenuate inflammation may subsequently decrease mortality.
